# The gut microbiota facilitate their host tolerance to extreme temperatures

**DOI:** 10.1186/s12866-024-03277-6

**Published:** 2024-04-20

**Authors:** Ziguang Wang, Yujie Wu, Xinxin Li, Xiaowen Ji, Wei Liu

**Affiliations:** 1 School of Plant Protection, Anhui Province Key Laboratory of Crop Integrated Pest Management, Anhui Province Engineering Laboratory for Green Pesticide Development and Application, Anhui Agricultural University, Hefei, China; 2 First Clinical Medical College, Mudanjiang Medical College, Mudanjiang, China; 3 China-Japan Union Hospital, Jilin University, Changchun, China

**Keywords:** Gut microbiota, Extreme temperatures, Tolerance, Insulin pathway, Carbohydrate metabolism

## Abstract

**Background:**

Exposure to extreme cold or heat temperature is one leading cause of weather-associated mortality and morbidity in animals. Emerging studies demonstrate that the microbiota residing in guts act as an integral factor required to modulate host tolerance to cold or heat exposure, but common and unique patterns of animal-temperature associations between cold and heat have not been simultaneously examined. Therefore, we attempted to investigate the roles of gut microbiota in modulating tolerance to cold or heat exposure in mice.

**Results:**

The results showed that both cold and heat acutely change the body temperature of mice, but mice efficiently maintain their body temperature at conditions of chronic extreme temperatures. Mice adapt to extreme temperatures by adjusting body weight gain, food intake and energy harvest. Fascinatingly, 16 S rRNA sequencing shows that extreme temperatures result in a differential shift in the gut microbiota. Moreover, transplantation of the extreme-temperature microbiota is sufficient to enhance host tolerance to cold and heat, respectively. Metagenomic sequencing shows that the microbiota assists their hosts in resisting extreme temperatures through regulating the host insulin pathway.

**Conclusions:**

Our findings highlight that the microbiota is a key factor orchestrating the overall energy homeostasis under extreme temperatures, providing an insight into the interaction and coevolution of hosts and gut microbiota.

**Supplementary Information:**

The online version contains supplementary material available at 10.1186/s12866-024-03277-6.

## Background

Temperature acts as the most critical abiotic factor that profoundly affects many aspects of physiology in animals [1]. Currently, dramatic temperature fluctuation causes extreme summer hotness and winter cold in many regions. Moreover, global temperature regimes are expected to continue rapid shifts in the future [2], yet we lack an understanding of how climate change predictably influences thermal physiology and tolerance [3]. Humans can live in zones of extreme temperatures generally ranging from − 50 °C to + 50 °C, so this limited tolerance to extreme temperatures frequently accounts for morbidity and mortality in life [4]. Exposure to extreme heat usually affects vulnerable populations of many animal species in low-latitude regions, while exposure to low temperature is more prevalent in the global biosphere (∼85%), including high-altitude zones and deep oceans [5]. Sudden temperature changes catastrophically threaten biodiversity in the ecosystem as well [6]. Therefore, adaptation to extreme temperatures is necessary for all species ranging from bacteria to mammals [7, 8]. Animals modulate their food intake and energy demand under the condition of extreme-temperature stress [9]. On the molecular level, cells adjust to gradual changes in temperature by modifying gene expressions associated with membrane fluidity and conformational flexibility [10]. For instance, cells can express cold-shock proteins, antifreeze proteins, and cryoprotectants that protect them from freezing when challenged with cold stress [11]. Extensive studies have revealed aspects of animal physiology in response to extreme temperatures, but our knowledge of the underlying mediators and mechanisms remains poor.

The gastrointestinal tract of mammals routinely harbors the densest communities of microbes that fundamentally affect host metabolism, immunity, and neurobehaviors [12–14]. Emerging studies show that animal tolerance to extreme temperatures is closely associated with the microbiota in the intestine. The gut microbiota of many animals is also affected by temperature [15–17]. For example, the long-term heat exposure elevated the relative abundance of *Lactobacillus* and *Oscillospira*, but reduced that of *Blautia* and *Allobaculum* in the intestine microbiota of rats [15]. Additionally, warm-reared hosts are prone to harbor higher relative abundances of pathogenic Mycobacterium and Proteobacterium [18]. On the other hand, studies found that the microbiota plays an important role in regulating host tolerance to cold stress [12, 19]. Unfortunately, most studies have separately identified cold and heat as contributing factors affecting thermal physiology, but a universal signature of temperature-associated microbiota has not been identified. Given both cold and heat can give rise to weather-associated mortality and morbidity [20], common and unique patterns of animal-temperature associations across a range of temperatures urgently need to be explored.

With a surge of interest that microbiome shapes many aspects of host physiology in a wide diversity of species, we attempted to investigate microbiome roles in modulating tolerance to cold or heat exposure in mice. We comprehensively characterize associations of extreme cold and heat with animal pathophysiology. We found that extreme temperatures chronically affected the weight body gain and food intake. Interestingly, both cold and heat changed the composition and function of the microbiota, consequently contributing to host fitness in response to ambient temperature. Therefore, our findings that a comprehensive overview of extreme-associated microbiota facilitates understanding of animal-microbe mutualism in conditions of climate fluctuation, providing insight into the strategy to predict and mitigate the effects of global changes on wildlife and human.

## Methods

### Animals

Animal experiments were approved by the Institutional Animal Care and Use Committee of Anhui Agricultural University (Anhui, China). All C57Bl/6J wild-type adult male mice came from Shanxi Medical University. Acclimatized animals based on their body weights (18–24 g) were randomly assigned to each cage containing 2 animals. Mice were raised with a standard chow diet (16.2 MJ/kg Gross Energy; 9 kJ% Fat, 33 kJ% Protein, 58 kJ% Carbohydrates) in 12 h day and night cycles unless otherwise stated. Each group of mice had *ad libitum* access to a standard chow diet and water. Room temperature, cold and heat exposures were set at 25 , -5 °C, and 35 °C in climatic chambers with individually ventilated cages. The experiment lasted for 60 days and then feces were collected for 16S rRNA sequencing. The experiment schematic is illustrated in Fig. S1.

For depletion of microbiota, fresh antibiotics were daily freshly prepared and administered in the drinking water *ad libitum* [21] containing 100 µg/ml Neomycin, 50 µg/ml Streptomycin, 100 U/ml Penicillin, 50 µg/ml Vancomycine, 100 µg/ml Metronidazole, 1 mg/ml Bacitracin, 125 µg/ml Ciprofloxacin, 100 µg/ml Ceftazidime and 170 µg/ml Gentamycin (when longer than 60 days treatment). All antibiotic treatments started at 8 weeks old animals. Microbiota transplantations were performed by gavage of 20 mg fresh feces resuspended in 400 µl sterile anaerobic 1×PBS [22]. Mice were euthanized by cervical dislocation after treatment.

### Body mass and temperature assay

We repeatedly measured their body mass by platform balance on a daily basis of the acclimation period. The rectal temperature assay was read with a contact thermometer (TES-1310).

### Hepatic glycogen and triglycerides content

Each 10 mg liver tissue taken from -80 °C was homogenized with 200 µl 30% KOH on ice and the homogenate was boiled for 10 min to inactivate enzymes. The boiled samples were centrifuged at 12,000 rpm for 15 min at 4 °C to remove insoluble materials and the supernatant was ready for the assay using a Glycogen Assay Kit (colorimetric) (Sigma), and the results were normalized to the weight of initial tissues. 500 µl of blood was taken from terminally anesthetized mice in tubes with 15 ul of 0.5 mM EDTA, 4 µl of aprotinin (1.3%) and 4 µl of DPP-IV (10mM) and plasma stored at -80 °C. Triglycerides were measured by Triglycerides Assay Kit (Sigma).

### Hematoxylin and eosin (H&E) staining and adipocyte size measurements

Adipose tissues and duodenum were rinsed with saline solution to remove blood, fixed in 10% neutral formalin buffered solution, embedded in paraffin, and sectioned at 4–5 μm thickness with a microtome [23]. The samples were stained with hematoxylin-eosin (H&E) using standard techniques [24]. Adipocyte cell sizes were assessed as described previously. Briefly, images were captured using a light microscope equipped with a digital camera (Leica). Three to five scopes per slide were chosen randomly by one blinded evaluator. Cell sizes were analyzed using ImageJ software.

### Fecal microbiota transplantation

The mice were allowed to grow to 8 weeks of age and were given 8 kinds of antibiotics prepared and added to the drinking water. To deplete the microorganisms in the intestines of mice, antibiotics were fed to mice for two months. The total number of bacteria was tested before microbial transplantation. The total bacterial load was dramatically reduced to 0–46 CFUs per gram in feces by plating the diluted feces on nutrient agar plates. Fresh feces were taken from RT-, cold-stimulated and heat-stimulated mice for up to 60 days, respectively. Weighing of fresh feces from mice using an electronic balance. Feces were diluted 10-fold in PBS in a sterile environment and the fecal residue was filtered after grinding. The suspension was placed in a centrifuge at 900 rpm/min. Centrifuge for 3 min, repeat twice and discard the precipitate, which is the purified fraction [25, 26]. Daily FMT using purified fresh fecal fluid.

### 16 S rRNA gene and metagenomic sequencing

Cecal content samples were collected into clean Eppendorf tubes and stored at -80 °C after snap freezing in liquid nitrogen [27]. Bacterial genomic DNA was extracted using QIAamp Fast DNA stool Mini Kit (Qiagen). Bacterial DNA was PCR amplified with barcoded universal bacterial primers (515 F:5-GTGCCAGCMGCCGCGGTAA-3, 806R:5-GGACTACHVGGGTWTCTAAT-3) targeting variable regionV4 of 16SrRNA gene. Pyrosequencing (Novogene, Beijing, China) was conducted with Illumina MiSeq 2 × 250 platforms. The sample sequence reads were processed using QIIME 2.0 using the parameters. For each dataset, demultiplexed sequencing reads were denoised to generate amplicon sequence variants (ASVs) using DADA2 algorithm [28]. Additional parameters were used to denoise dataset, according to DADA2 protocol [29]. Species annotation was performed using the reference database (Silva database, https://www.arb-silva.de/). The Shannon index and observed species were computed to assess the alpha diversity. The principal component analysis (PCA) was based on weighted unifrac analysis of ASVs to assess beta diversity and then plotted with ggplot2 package. The heatmap, which visually displays different abundance and taxa clustering, is drawn by the abundance information of top 35 taxa of each sample at Phylum and Genus. Permutational multivariate analysis of variance (PERMANOVA) was used to evaluate the composition and structure of bacterial profiles among different groups [30].

Metagenomic sequencing and general data analyses were performed by Shanghai Majorbio Bio-pharm Biotechnology (Shanghai, China). A library composed of 400-bp clone inserts was generated for associated samples. The clean reads of metagenomic sequencing were assembled to generate long contig sequences using the SOAPdenovo assembler. Several Kmer frequencies were applied to generate different assembly results, and N50 lengths were utilized to generate optimal assembly results [31]. The assembled sequences of each sample were used as input files in the next MG-RAST analysis [32]. To minimize the potential effect of differences in sequence processing between data sets, we ran all metagenomics sequences through the MG-RAST pipeline (e.g. gene and protein features prediction) using default parameters. The Principal Component Analysis (PCA) was analyzed to evaluate the most representative organisms of metagenomics. Linear discriminant analysis Effect Size (LefSe) was used to determine the significant difference in the abundant feature among groups [33]. Due to the different sequencing depths, we used MG-RAST to estimate normalized abundances of metabolic genes and pathways of KEGG and taxon under default parameters. We also performed Carbohydrate-active enzymes (CAZyme) and Clusters of Orthologous Groups (COG) function annotations using the CAZy database and eggNOG database.

### Real-time qPCR analysis

The jejunum and fat pad were dissected in cold PBS buffer, and overall RNA was extracted with Trizol reagent (Invitrogen, USA). Up to 2 µg overall RNA was used for cDNA arrangement with arbitrary hexamer primers using a High Capacity cDNA Reverse Transcription kit (Applied Biosystems). Homeostasis mRNA interpretation was defined by qualitative real-time PCR using the Light Cycler 480 SYBR Green Master I Mix (Roche) with 386 well Light Cycler 480 II (Roche). Transcription levels were Standardized to the meaning relative expression of both acidic ribosomal phosphoprotein 36b4 (gene Rplp0) and small ribosomal protein 16 (Rps16) or to beta-2-microglobulin (B2m) for fatty tissue, as indicated [34]. The relative expression value was computed with formula: △Ct = Ct (target gene) – Ct (reference gene), the relative = 2^−△△Ct^. The primer sequences used for the real-time PCR are shown in supplementary Table 1.

### Statistics

Statistical analysis is performed using GraphPad Prism 9.0 and indicated inside each figure legend. The layout of all figures used Adobe Illustrator 2022. A two-tailed Student’s t-test was used to compare two groups, and one-way ANOVA followed by Tukey’s multiple comparisons test was used for comparisons of multiple groups. All experiments were performed at least three times, and the representative experiments are shown. All data are presented as the mean ± SEMs. No asterisk denotes *P* > 0.05; asterisk denotes *P* < 0.05; double-asterisk denotes *P* < 0.01; triple asterisk denotes *P* < 0.001.

## Results

**Fig. 1:**
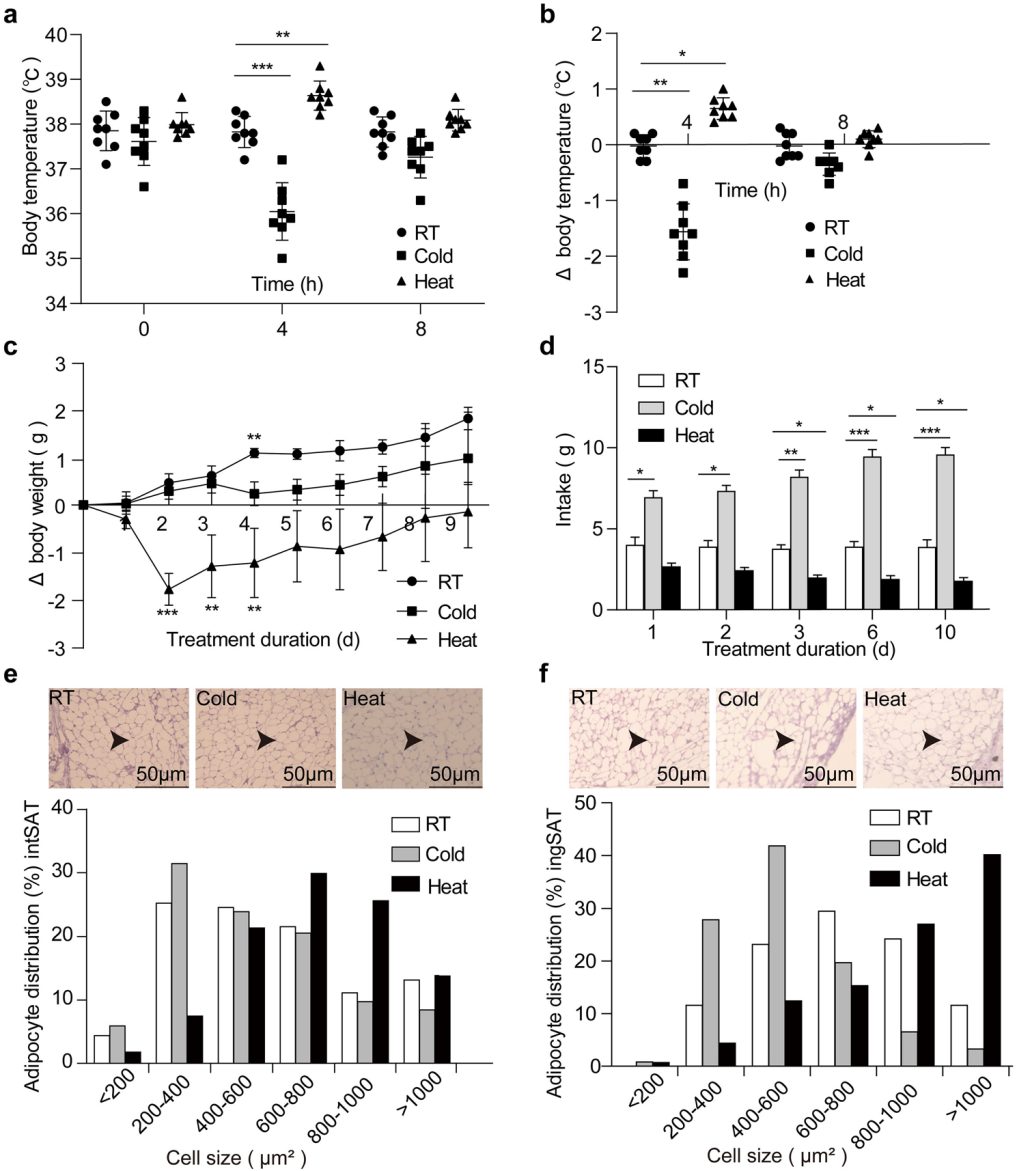
Temperature is a critical factor for body temperature maintenance and energy harvest. (**a**) The rectal temperature of mice after 4 and 8 h of room temperature (RT), cold temperature (Cold) and heat temperature (Heat). *n* = 8. (**b**) Changes in rectal temperature compared to initial as in (**a**). (**c**) Changes in body weight (compared to initial body weight) following RT, Cold and Heat. *n* = 8. (**d**) Food intake by mice was raised at RT, Cold and Heat. *n* = 8. They dramatically changed at the beginning of treatment, but gradually adapted to new stimuli. We only showed the results of the first 9 day in the Fig. 1c, d. (**e-f**) Hematoxylin-eosin (H&E) staining on paraffin sections from intSAT (**e**) and ingSAT (**f**) of RT, Cold and Heat mice. Cell size profiling of adipocytes from intSAT (**e**) and ingSAT (**f**) after 60 days of RT, Cold and Heat mice. The values show % from the total number of analyzed cells. Scale bars: 50 μm. All values show mean ± SEM. Significance was calculated using one-way ANOVA followed by Tukey’s multiple comparisons test. *p ≤ 0.05, **p ≤ 0.01, ***p ≤ 0.001

Extreme temperatures affect body temperature and energy harvest Environmental temperature acts as an important factor to influence physiology in animals. We addressed this issue by exposing C57Bl/6J adult mice to extreme cold and heat, respectively. Firstly, we found that mice succumbed to -10 °C and 37 °C within 1 day (Fig. S2a), so optimized − 5 °C and 35 °C as extreme cold and heat conditions for treatment, respectively. During the initial 4 h of acute chill exposure, our result showed that mice had a significant decrease in the rectal temperature compared to their room temperature (RT) counterpart (Fig. 1a, b). In contrast, heat-treated mice showed an increase in the rectal temperature. However, neither cold nor heat changed the rectal temperature compared to their RT counterparts after 8 h exposure (Fig. 1a, b), suggesting that mice adapted to acute extreme temperatures. Consistent with previous studies [35], long-term cold exposure modestly increased body weight gain compared to their initial body weight, whereas long-term heat exposure otherwise decreased it (Fig. 1c). Presumably, animals can maintain their body temperature by regulating compensatory metabolism to change caloric harvest from the diet [36]. Indeed, long-term cold exposure for up to 10 days increased food intake, while long-term heat exposure reduced food intake (Fig. 1d). These results suggest that animals change their feeding behavior and metabolism according to environmental temperature [37]. The adipocyte size distribution is related to the browning of the white fat depots. We found that cold-treated mice had elevated number of small and reduced number of large adipocytes in visceral depots of the white adipose tissue (intSAT and ingSAT, respectively) (Fig. 1e, f). In addition, the adipose depots from the cold-treated animals were darker in appearance. These suggest that chill increases the browning of the white fat depots. On the other hand, heat-treated mice exhibited a decreased number of small and increased number of large adipocytes in the corresponding positions. In conclusion, environmental temperature is a key factor that influences whole-body metabolism and energy harvest.

### Microbiota are required for the body temperature maintenance

**Fig. 2:**
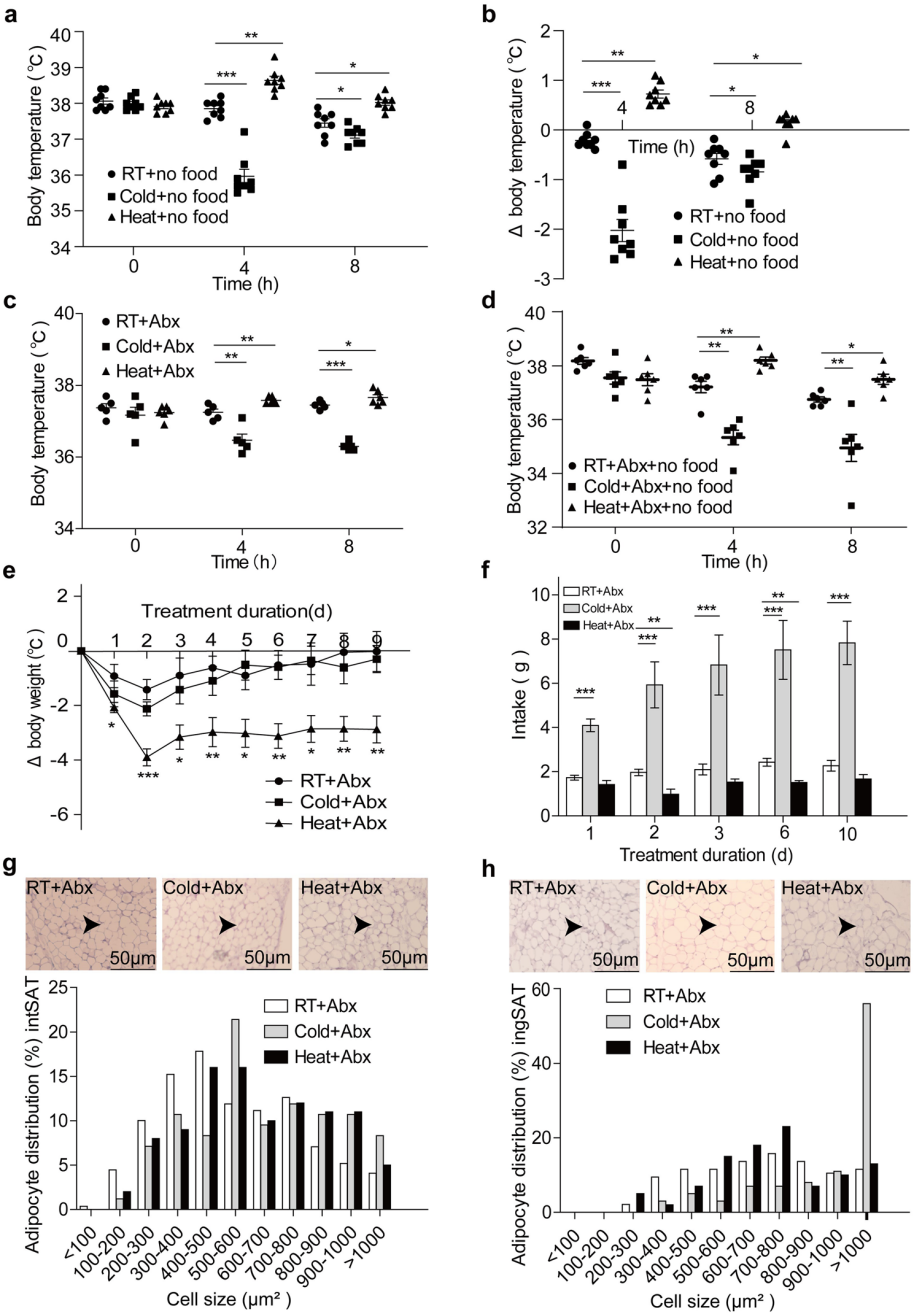
Intestinal microbiota are required for temperature maintenance. (**a**) The rectal temperature of mice treated with food fasting after 4 and 8 h of RT, Cold and Heat groups. *n* = 8. (**b**) Changes in rectal temperature compared to initial as in (**a**). (**c**) Changes in the rectal temperature of mice treated with antibiotics (Abx) after 4 and 8 h of RT, Cold and Heat groups. *n* = 6. (**d**) Changes in the rectal temperature of mice treated with Abx and food fasting after 4 and 8 h of RT, Cold and Heat groups. *n* = 6. (**e**) Changes in body weight of mice (compared to initial body weight) treated with Abx at RT, Cold and Heat. *n* = 5–6. (**f**) Food intake by mice treated with Abx at RT, Cold and Heat. *n* = 5–6. (**g**-**h**) H&E staining on paraffin sections from intSAT (**g**) and ingSAT (**h**) of RT + Abx, Cold + Abx and Heat + Abx mice. Cell size profiling of adipocytes from intSAT (**g**) and ingSAT (**h**) of mice treated with antibiotics after 60 days of RT + Abx, Cold + Abx and Heat + Abx mice. The values show % from the total number of analyzed cells. Scale bars: 50 μm. Significance was calculated using one-way ANOVA followed by Tukey’s multiple comparisons test. *p ≤ 0.05, **p ≤ 0.01, ***p ≤ 0.001

Alterations in food consumption were observed following exposure to extreme temperatures, prompting us to study whether food fasting could influence body temperature maintenance. Interestingly, starved mice still displayed a decrease in the rectal temperature at 8 h post cold exposure compared to *ad libitum*-fed control mice (Fig. 2a, b). Likewise, starved mice exhibited an increase in the rectal temperature at 8 h post heat exposure. These results suggested that energy restriction compromises body temperature maintenance. Because the diet is closely associated with the dynamics of intestinal microbiota, the findings provided a clue that the intestinal microbiota might play a role in body temperature maintenance. To this end, we depleted the intestinal microbiota with an antibiotics cocktail (Abx) in drinking water. Indeed, Abx-fed mice failed to maintain their body temperatures at 8 h post cold or heat exposure compared to *ad libitum*-fed control mice (Fig. 2c), indicating that the microbiota is required for tolerance to heat. Moreover, the combination of fasting and Abx aggravated the impairment of body temperature maintenance in cases of cold and heat exposure compared to conventionally reared (Fig. 2d and Fig. S2b). As expected, long-term heat exposure reduced body weight gain of Abx-treated mice (Fig. 2e). Intriguingly, our result showed that long-term cold exposure did not enhance body weight gain of Abx-treated mice, presumably due to the side effects of antibiotics on energy harvest. Consistently, long-term cold exposure for up to 10 days increased the food intake of Abx-treated mice (Fig. 2f), while long-term heat exposure modestly reduced their food intake. In addition, we found that Abx-treated mice with long-term cold exposure only had a mildly elevated number of small and reduced number of large adipocytes in visceral depots of the white adipose tissue (intSAT and ingSAT, respectively) (Fig. 2g, h). These results implicated that intestinal microbiota plays a critical role in tolerance to ambient extreme temperatures.

### Extreme temperatures change the composition of microbiota

**Fig. 3:**
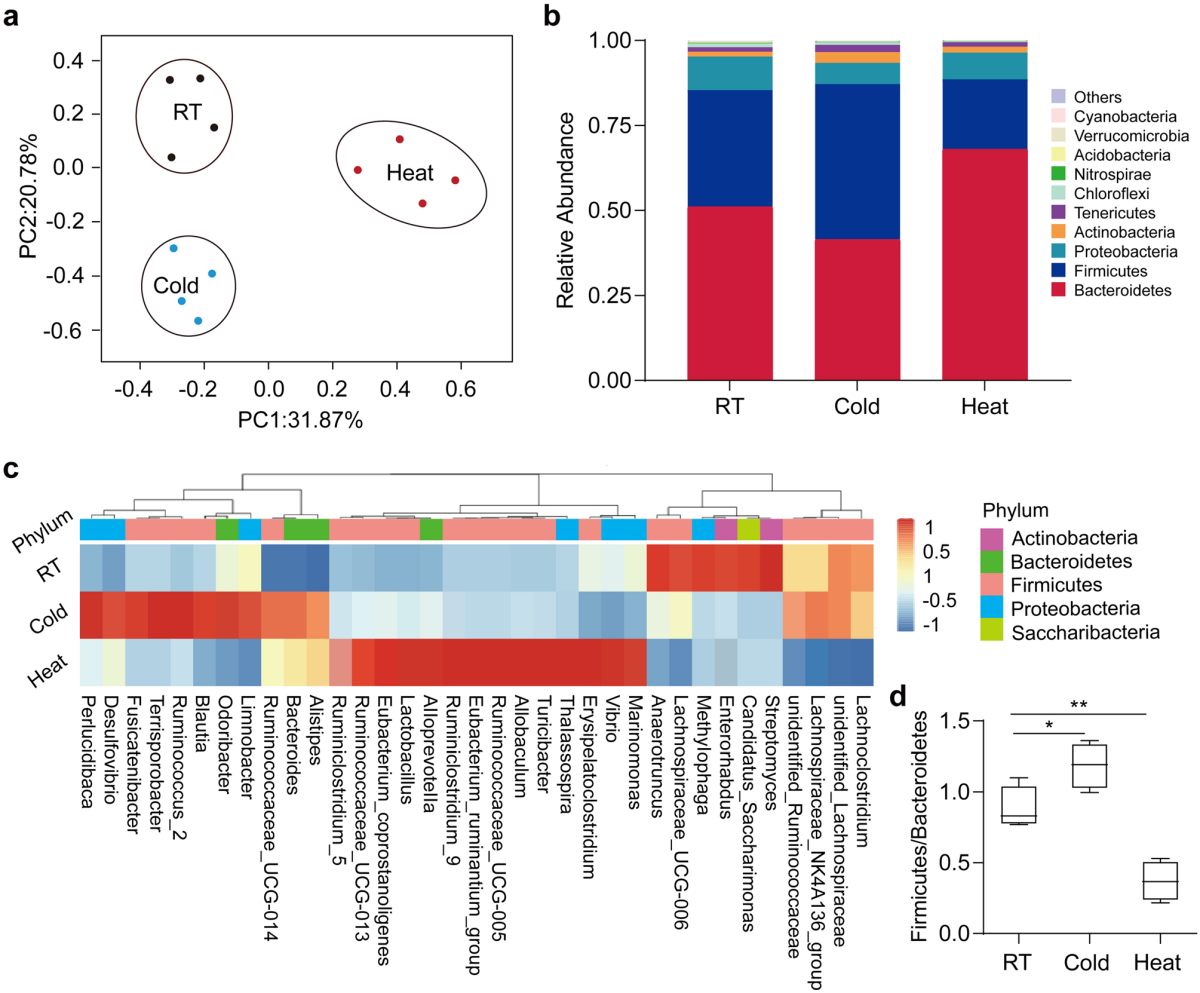
Long-term extreme temperature stress changes the gut microbiota composition. (**a**) Principal component analysis (PCA) based on weighted UniFrac analysis of ASVs. Each symbol represents a single sample of rectal after 60 days of RT, Cold and Heat. *n* = 4. (**b**) Comparison of phylum-level proportional abundance of the intestinal microbiota of up to 60 days RT, Cold and Heat mice. (**c**) Hierarchical clustering diagram using the average-neighbor (HC-AN) method comparing rectal of 60 days RT, Cold and Heat mice. Associated heatmap shows the relative abundance of representative ASVs selected for *p* < 0.05, obtained with a Welch t test comparison of the two groups and then grouped into families. The P value for the correlation between temperature and gut microbiota was corrected using a false discovery rate. One representative ASV with the greatest difference between the two group means from each family is selected for inclusion in the heatmap diagram. ASVs are shown as: Phylum and Genus. (**d**) Phylum level proportional abundance in rectal. *n* = 4. All values show mean ± SEM. Significance was calculated using non-paired two-tailed Student’s *t* test. *p ≤ 0.05, **p ≤ 0.01

To investigate whether prolonged extreme temperatures influenced the composition of intestinal microbiota, we collected the cecum content of post-mortem mice to profile the microbiota composition by sequencing the V4 region of 16 S rRNA gene. Fascinatingly, the principal component analysis (PCA) of weighted jack-knifed UniFrac distances of microbial communities to visualize inter-sample similarity showed that the gut microbiota within the cold, heat and RT mice were separately clustered (Fig. 3a), suggesting that cold or heat exposure leads to alterations in the composition of the microbiota. More importantly, microbiota composition from cold-exposed mice mainly differed from RT mice on the PC2 level, while microbiota composition from heat-exposed mice differed from control on the PC1 level. These results suggested that the microbiota undergo a distinct shift under conditions of cold and heat exposure. To decipher shifts in the microbiota composition, we examined differences in Amplicon Sequence Variants (ASVs) abundance of the ten richest groups on the phylum level, accounting for an average of 99.1% of the microbiota composition per sample. We found that Firmicutes is dominant (51.1%), and Bacteroides were secondary most abundant (34.3%) in mice raised at room temperature. The pronounced shifts of phylum-level proportional abundance were observed in both heat- and cold exposure, respectively. The relative abundance of Bacteroides predominantly increased to 68.0% of total ASVs following chronic heat treatment, while the relative abundance of Firmicutes decreased to 20.6% of ASVs. In contrast, the dominant phylum of Bacteroides was replaced by Firmicutes (45.7%) after chronic cold treatment (Fig. 3b). These results indicate the reverse direction in a shift of phylum-level proportional abundance in cold and heat exposure. Interestingly, heat reduced alpha diversity of gut microbiota in terms of Shannon index, but cold did not (Fig. S2d). Next, we determined the relative abundance of top ASVs in detail. Comparison of phylum level proportional abundance showed shifts in proportions of Firmicutes and Bacteroidetes, especially in the ratio Firmicutes/Bacteroidetes, in cold- and heat-exposure. In the case of cold exposure, Firmicutes abundance (from 34.3% in RT up to 45.7%) increased over Bacteroidetes (from 51.1% in RT to 41.4%), and became the most abundant phylum (Fig. S2f, g). As opposed to the observations in cold mice, Bacteroidetes (68.0%) were increased upon heat stress, while Firmicutes (20.6%) were decreased (Fig. S2f, g). Therefore, the reverse shifts of the Firmicutes/Bacteroidetes ratio were observed in cold- and heat-exposure groups compared to RT controls (Fig. 3d). However, those within Proteobacteria and Actinobacteria in either cold- or heat-treated mice were basically comparable to RT controls (Fig. S2h, i). Finally, we further examined individual genus, or family-based hierarchical clustering using the average-neighbor method. The result confirmed the major shift of the microbiota composition in the cold- or heat-exposed versus RT groups (Fig. 3c). Collectively, these results demonstrate that extreme temperatures altered the composition of gut microbiota in mice.

### Microbiota promote the tolerance to extreme temperatures

**Fig. 4:**
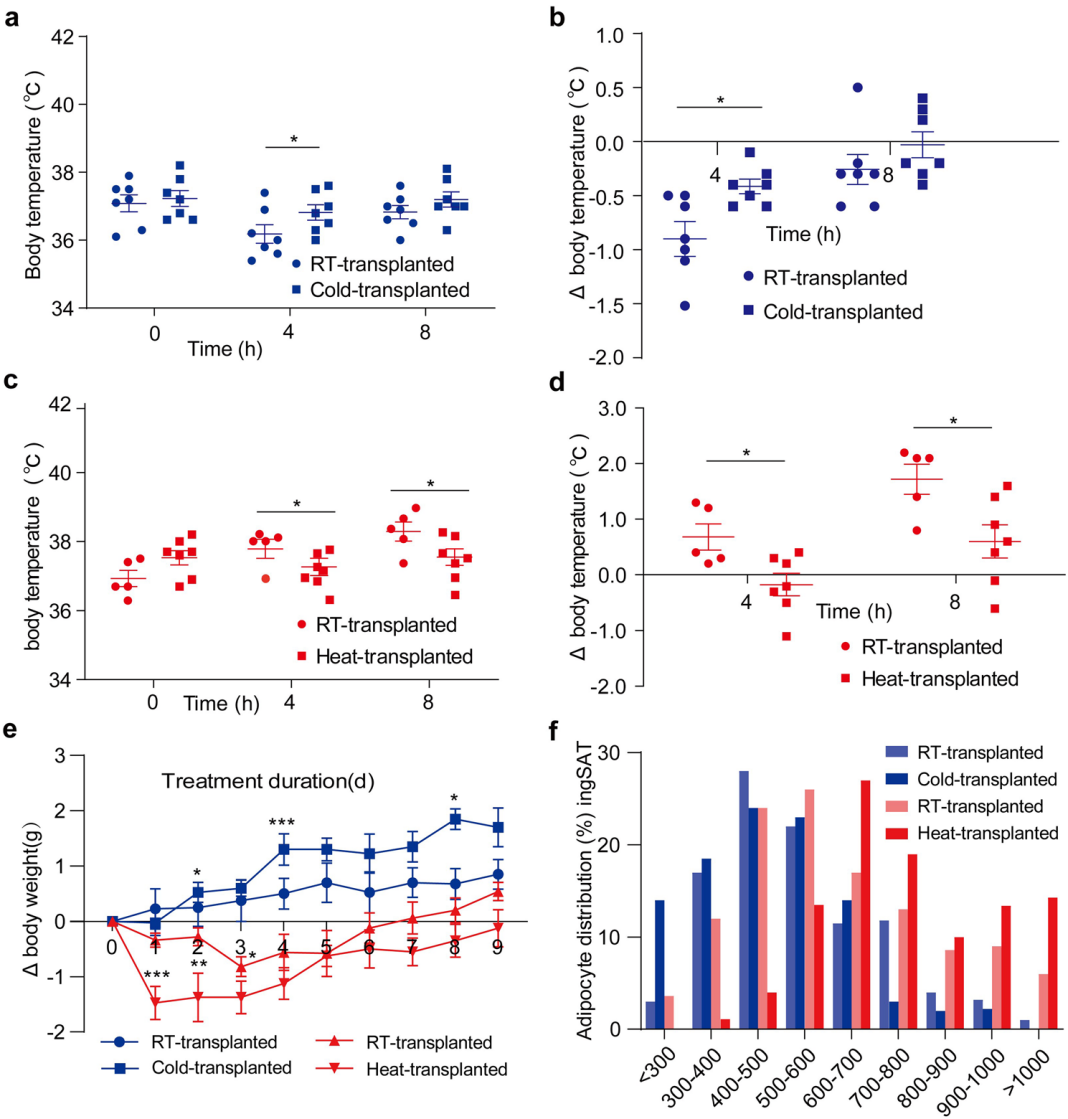
Extreme temperature microbiota enhances the tolerance to extreme temperatures. (**a**) The rectal temperature changes of RT and Cold microbiota-transplanted mice after 4 and 8 h of cold stress. *n* = 7. (**b**) Changes in temperature change of RT and Cold microbiota-transplanted mice after 4 and 8 h of heat stress compared to initial as in (**a**). (**c**) The rectal temperature change of RT and Heat microbiota-transplanted mice after 4 and 8 h of heat stress. *n* = 5–7. (**d**) Changes in temperature change of RT and Heat microbiota-transplanted mice after 4 and 8 h of heat stress compared to initial as in (**c**). (**e**) Changes in body weight of mice transplanted with RT-, Cold- and Heat-microbiota. *n* = 5–7. (**f**) Cell size profiling of adipocytes from ingSAT after 60 days of RT, Cold and Heat microbiota-transplanted mice. The values show % from the total number of analyzed cells. The blue is at cold exposure (-5°C), and the red is at heat exposure (35°C). All values show mean ± SEM. Significance was calculated using non-paired two-tailed Student’s *t* test. *p ≤ 0.05, **p ≤ 0.01, ***p ≤ 0.001

To investigate the causal role of microbiota in regulating tolerance to extreme temperatures, we transplanted the microbiota from cold-exposure, heat-stress and control mice to Abx-treated mice. First, we examined the rectal temperature of microbiota-transplanted mice after 4 and 8 h exposure to extreme temperatures. In response to cold exposure, recipients of cold microbiota exhibited less decrease in rectal temperature following 4 h of cold-exposed than recipients of RT microbiota (Fig. 4a, b). These results indicate that the intestinal microbiota is a prerequisite for tolerance to acute cold stress. It was likely that cold microbiota modestly improved host tolerance to long-term cold stress. On the other hand, our data showed that recipients of heat microbiota exhibited decreased temperature following both 4 and 8 h heat stress compared to recipients of RT microbiota (Fig. 4c, d). Together, these results suggest that the microbiota are sufficient to facilitate host tolerance to extreme-temperature stress. Consistently, changes in the microbiota composition can lead to dysfunction of the microbiota, which consequently influences the health and disease of hosts [38, 39]. As expected, we found that transplantation with heat microbiota resulted in a dramatic loss of body weight within the first 3 days in the RT condition, but transplantation with RT microbiota still had a stable body weight (Fig. 4e), indicating that the heat microbiota affects the loss of body weight in the presence of heat. Interestingly, we did not find any difference in food intake (Fig. S2c), suggesting that heat-microbiota only affect the efficiency of energy harvest. By contrast, transplantation with cold microbiota apparently elevated body weight in the cold condition (Fig. 4e). To further investigate whether extreme temperatures microbiota could alter the browning process, we examined the adipocyte size of ingSAT depots. The result showed that heat-transplanted mice exhibited an increased number of large unilocular adipocytes. Inversely, an increased number of small adipocytes was observed in cold-transplanted mice (Fig. 4f). Together, these results indicate that heat or cold microbiota are sufficient to affect energy dissipation by changing beige.

### Extreme-temperature microbiota affect intestinal morphology

**Fig. 5:**
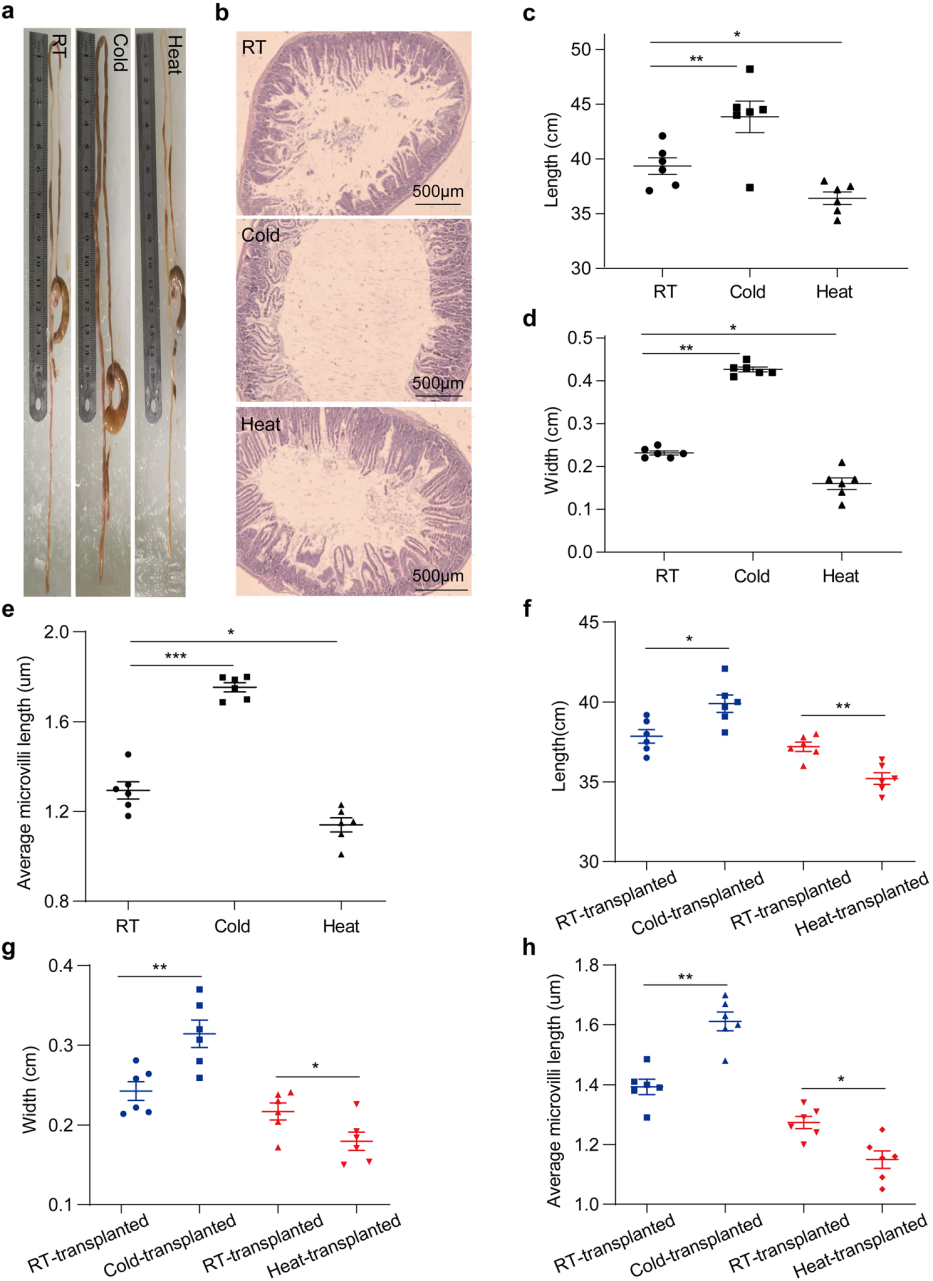
Cold and heat microbiota-transplanted mice altered intestinal morphology. (**a**) Representative images of the cecum, small intestine, and colon of RT, Cold and Heat mice. (**b**) H&E staining of duodenum of RT, Cold and Heat mice. Scale bars: 500 μm. (**c**) Small intestine lengths of 60 days RT, Cold and Heat mice. *n* = 6. (**d**) Small intestine widths of 60 days RT, Cold and Heat mice. *n* = 6. (**e**) Average microvilli lengths of RT, Cold and Heat mice. (**f**) Small intestine lengths of 60 days RT, Cold and Heat microbiota-transplanted mice. *n* = 6. (**g**) Small intestine widths of 60 days RT, Cold and Heat microbiota-transplanted mice. *n* = 6. (**h**) Average microvilli lengths of RT, Cold and Heat microbiota -transplanted mice. *n* = 6. The blue is at cold exposure (-5°C), and the red is at heat exposure (35°C). All values show mean ± SEM. Significance was calculated using non-paired two-tailed Student’s t test and one-way ANOVA followed by Tukey’s multiple comparisons test. *p ≤ 0.05, **p ≤ 0.01, ***p ≤ 0.001

Food intake and body weight gain result from food digestion and absorption in the intestine, prompting us to investigate the effect of extreme temperatures on the morphology of the intestine. Indeed, we observed an evident increase in the lengths and widths of the small intestine in the cold-exposure mice for up to 60 days (Fig. 5a-d). Furthermore, the length of villi was increased following the exposure to cold (Fig. 5e). Conversely, a marked decrease in the lengths and widths of the small intestine was observed in heat-treated mice. At the same time, the length of villi was decreased following the exposure to heat. These results suggested the plasticity of small intestine responding to the temperature-induced energy demand. Given the changes in the small intestine morphology, we attempted to exploit it following microbiota transplantation. As expected, cold microbiota-transplanted mice exhibited a remarkable increase in intestinal lengths and widths compared to the RT microbiota-transplanted control (Fig. 5f, g). In addition, the length of villi was enhanced in cold microbiota-transplanted mice (Fig. 5h). Conversely, heat microbiota-transplantation contributed to a decrease in the intestinal lengths and widths compared to the RT microbiota-transplanted control. Indeed, the length of villi was reduced in heat microbiota-transplanted mice. Altogether, these results show that cold or heat microbiota were sufficient to change intestinal morphology.

### Microbiota facilitate host tolerance via insulin pathway

**Fig. 6:**
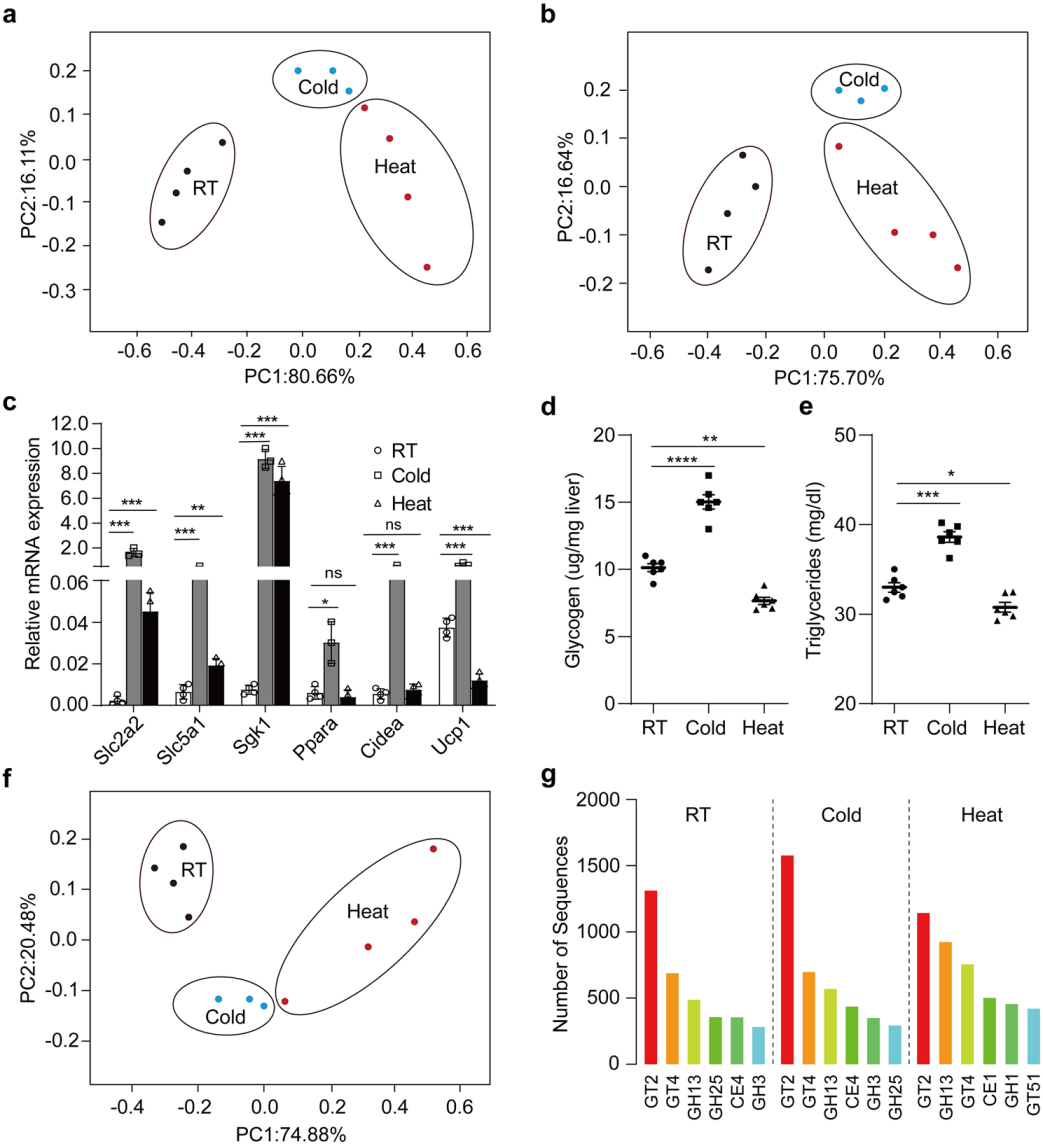
Extreme temperature microbiota affected insulin sensitivity and carbohydrate-active enzymes. (**a**) Principal component analysis of the composition of intestinal microbiota based on metagenomic sequencing of cecal content samples at RT, Cold and Heat mice. *n* = 3–4. (*b*) Principal coordinates analysis of microbial functions associated with insulin pathway based on metagenomic sequencing of cecal content samples. *n* = 3–4. (**c**) Relative mRNA expression of host insulin pathway in the proximal cecum of RT, Cold and Heat mice quantified by real-time qPCR in glycometabolism (*Slc2a2, Slc5a1, Sgk1*) and lipid metabolism (*Ppara, Cidea, Ucp1*). (**d**-**e**) Concentrations of hepatic glycogen (**d**) and plasma triglyceride (**e**) of RT, Cold and Heat mice. *n* = 6. (**f**) Principal coordinates analysis of carbohydrate-active enzymes based on metagenomic sequencing of cecal content samples. *n* = 3–4. (**g**) Statistical chart of carbohydrate-active enzymes classification in RT, Cold and Heat mice. All values show mean ± SEM. Significance was calculated using one-way ANOVA followed by Tukey’s multiple comparisons test. *p ≤ 0.05, **p ≤ 0.01, ***p ≤ 0.001

To decipher alterations in functions of gut microbiota under the conditions of chronic extreme temperatures, we performed metagenomic sequencing of cecal content samples after mice were treated for up to 8 weeks. The PCA showed that the three conditions were substantially different, and the replicates were very similar within each condition (Fig. 6a), giving rise to a remarkable differentiation and supporting further analysis. Namely, heat induced the separation to the control on the first principal component of variance, while cold induced the separation to the control on the second principal component of variance. These results suggest that extreme temperatures play a critical role in regulating the composition of intestinal microbiota. Similar changes were observed in the three conditions with LDA Effect Size analysis (Fig. S2e). Next, we mapped the metagenomic reads to a gut microbial gene catalogue, evenly dividing reads mapping to more than one gene, and grouping genes into KEGG pathways. The top predicted functions were mainly involved in metabolism, genetic and environmental information processing and cellular processes at the 1st level. However, we did not observe an apparent difference in the 1st level pathways identified among the three groups (Fig. S3a). To further narrow down changes in metagenomic functions, we focused on microbial predicted functions that were associated with host insulin signal using the 3884 reads, because microbial metabolites, such as acetate and butyrate, stimulated insulin secretion [25, 40]. We, subsequently, analyzed activity differences of insulin signals among three groups with PERMANOVA revealed mice in the cecal content samples. Indeed, the PCA score plot with the top 10 alterations associated with insulin secretion showed a clear demarcation among RT, cold and heat groups (Fig. 6b). The pathway that was associated with host “Insulin secretion” was upregulated in the cecal content samples of cold mice compared to that of RT counterparts, whereas it was downregulated in heat mice. For instance, CDP-glucose 4, 6-dehydratase (positive for insulin signal) dropped in heat exposure, while glucose-6-phosphate isomerase (negative for insulin signal) rose. It was deduced that host insulin signal could be influenced when they were exposed to extreme temperatures. In this regard, the expression of insulin signal-associated genes was examined in the host cecum by quantitative PCR. Indeed, our data showed that expression levels of most genes were decreased after heat exposure (Fig. 6c), while they were enhanced after cold exposure. More importantly, levels of both glycogen and triglyceride were reduced after heat exposure (Fig. 6d, e), while they were increased after cold exposure. In addition, carbohydrate metabolism was analyzed in cecal content samples using the CAZy database. The PCA showed that RT, cold and heat three groups apart from each other (Fig. 6f), suggesting that extreme temperatures play roles in regulating the carbohydrate metabolism of gut microbiota. A total of 53 target genes were identified to function in the digestion and absorption of glucose, hydrolysis and synthesis of polysaccharides. Indeed, we observed significant differences among three groups of samples based on the profile of their carbohydrate-active enzymes (Fig. 6g), indicating that extreme temperatures affect microbial enzymes involved in carbohydrate metabolism. Intriguingly, COG function classification showed the modest segregation in energy production and conversion among three samples (Fig. S3b), but cold microbiota still had a higher energy production and conversion. Overall, these results suggested that the microbiota is required for host tolerance to extreme temperatures through regulating the host insulin signal pathway.

## Discussion

The frequency and magnitude of temperature fluctuations are expected to exasperate in the next century in the world [3]. Predicting microbial diversity responses to changing temperature regimes first requires an understanding of the mechanisms by which temperature impacts organismal physiology and fitness. In this study, we report that both cold and heat acutely change the body temperature of mice. During the initial 4 h of acute cold or heat exposure, mice displayed a significant decrease or increase in the rectal temperature. Consistent with previous studies [35], ambient temperature acutely changes the body temperature of mice, mirroring that temperature biology and clinical symptoms in human. Variation in environmental temperature is ubiquitous, making organisms endure to the extent extreme temperatures to ensure their survival and reproduction [41, 42]. We found that mice maintain their body temperature under conditions of chronic temperatures ranging from − 5 °C to 35 °C by adjusting body weight gain, food intake and energy assimilation. For example, higher body temperature increases peripheral blood flow and accelerates heat dissipation, consequently boosting their tolerance to heat stress [43]. Together, temperature has a significant effect on thermal physiology and animals adapt to changing temperature by modulating their metabolism and behaviors [44].

Recently, it has become clear that microbiota is important for the survival, homeostasis and development of animals [45, 46]. Extreme temperatures can shape the composition of the microbiota across many animal species [47–49]. In mammals, exposure to cold results in shifts in the community composition of gut microbiota with marked impacts on overall energy homeostasis [50]. In ectotherms, little increase in temperature resulted in impaired diversity and altered composition of microbiota in lizards and salamanders, which was correlated with decreased host survival [16, 17]. Therefore, temperature is virtually a major determinant of microbial diversity. We found that heat temperature could reduce alpha diversity of gut microbiota, but cold did not affect it. These changes in gut microbiota favored energy extraction and improved the host’s resistance to extreme temperatures, and microbiota transplantation enhanced the host’s tolerance to extreme temperatures. Microbial diversity is the consequence of co-evolution between microbial communities and hosts [51]. It’s assumed that this co-evolution allows for maximized uptake of calories from the consumed diet in case of elevated energy demand. In contrast, it can restrict food intake in case of dampened energy demand [52].

Nowadays, joint analyses of high-throughput human multi-omics data, including metagenomics, transcriptomics and metabolomics data, together with measures of host physiology provide insight into potential molecular mechanisms of phenotypes. Using metagenomics data, we found that the microbiota assists host in adapting to extreme temperatures through regulating host’s insulin pathway. The early study found that acetate and butyrate, generated by gut bacteria, improved glucose homeostasis by inducing the generation of glucagon-like peptide-1 and peptide YY, which in turn stimulated insulin secretion [25]. Interestingly, *Lachnospiraceae* which were involved in carbohydrate metabolism and butyrate production were increased upon cold stress (Fig. S2e) [53]. Moreover, altered insulin signal pathway were verified in hosts when exposed to extreme temperatures (Fig. 6c-e), suggesting that the gut microbiota facilitated host tolerance through altering insulin pathway. The alterations in carbohydrate metabolism of gut microbiota underlie the altered host insulin signals when exposed to extreme temperatures (Fig. 6f, g). Consistently, the latest studies show that gut microbial carbohydrate metabolism can contribute to insulin resistance [40]. In addition to the control of carbohydrate metabolism, insulin can regulate lipid metabolism by stimulating lipid synthesis in liver and fat cells and by attenuating lipolysis to fatty acids. Nevertheless, further studies to investigate this molecular mechanism that microbiota regulates the lipid metabolism of hosts at extreme temperatures.

Given global climate change exasperates, we attempted to investigate the potent roles of the microbiota in enhancing host tolerance to extreme temperatures. With a surge of interest in microbiota, we highlighted the improved tolerance to extreme temperatures following microbiota transplantation. Future studies to explore this molecular mechanism would improve the knowledge of host-microbiota interactions, providing an insight into the potential application of microbiota transplantation to patients subject to extreme temperatures.

## Conclusions

In our work, we provide an insight into the natural phenomenon that temperature acts as a critical factor to acutely affect the body temperature, but mice efficiently maintain body temperature in response to chronic extreme temperatures. We found that extreme temperatures result in a shift of the gut microbiota, and transplantation of the microbiota is sufficient to enable tolerance to heat and cold. Metagenomic sequencing shows that the microbiota assists hosts in adapting to extreme temperatures through regulating the host insulin pathway. In conclusion, our finding revealed that the microbiota is a key factor orchestrating the overall energy homeostasis under extreme temperatures, opening the door to a better understanding of the ecological relationships between the microbial and hosts.

### Electronic supplementary material

Below is the link to the electronic supplementary material.

Supplementary Material 1

## Data Availability

Raw sequence data is available under the NCBI Sequence Read Archive (SRA): SRR24755639-24755644 https://www.ncbi.nlm.nih.gov/sra/?term=SRR24755639. Further information and requests for resources and reagents should be directed to and will be fulfilled by the lead contact, Dr. Wei Liu (liuwei5@ahau.edu.cn).

## Abbreviations

Abx: antibiotics cocktail
H&E: Hematoxylin-eosin
RT: room temperature
Cold: cold temperature
Heat: heat temperature
intSAT: interscapular subcutaneous white adipose tissue
inGSAT: inguinal subcutaneous white adipose tissue
LEfSe: LDA Effect Size
PERMANOVA: Permutational multivariate analysis of variance
