# Supplementary material for: The gut microbiota facilitate their host tolerance to extreme temperatures
Source: BMC Microbiol. 2024 Apr 20;24:131. doi: 10.1186/s12866-024-03277-6 (PMC11031955; doi:10.1186/s12866-024-03277-6)
Supplement: Supplementary file 1 — Supplementary Material 1 [file 12866_2024_3277_MOESM1_ESM.docx]

Supplementary Figures


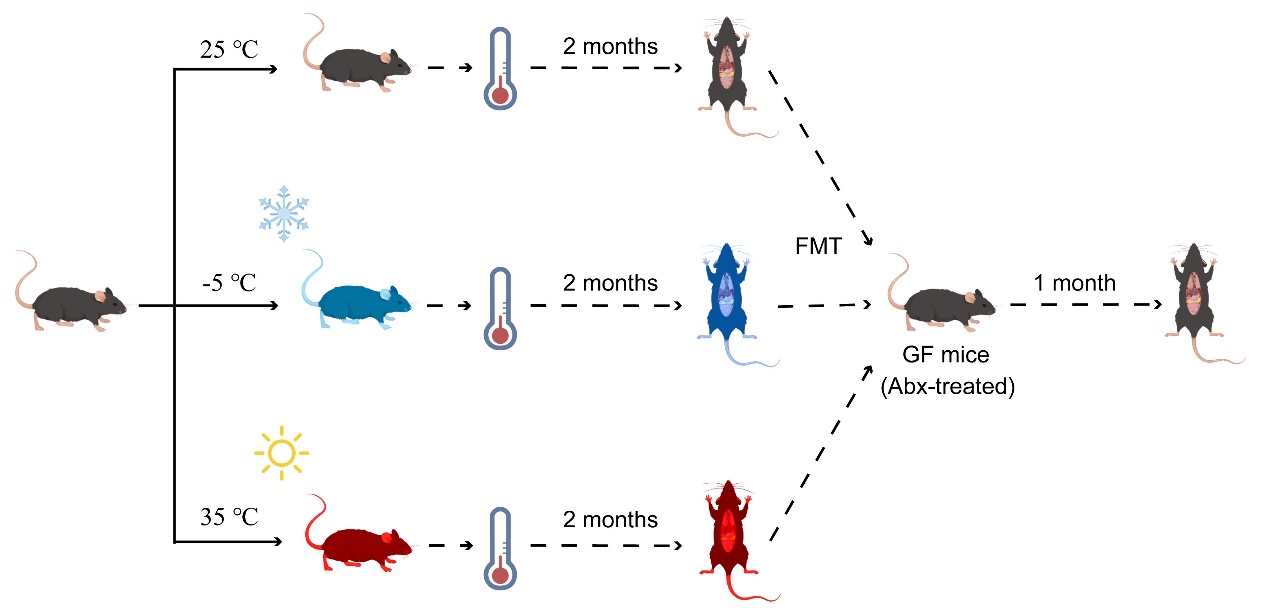


**Fig. S1** The schematic of experiment design. Room temperature, cold and heat exposures were set at 25℃, -5°C, and 35°C, respectively.


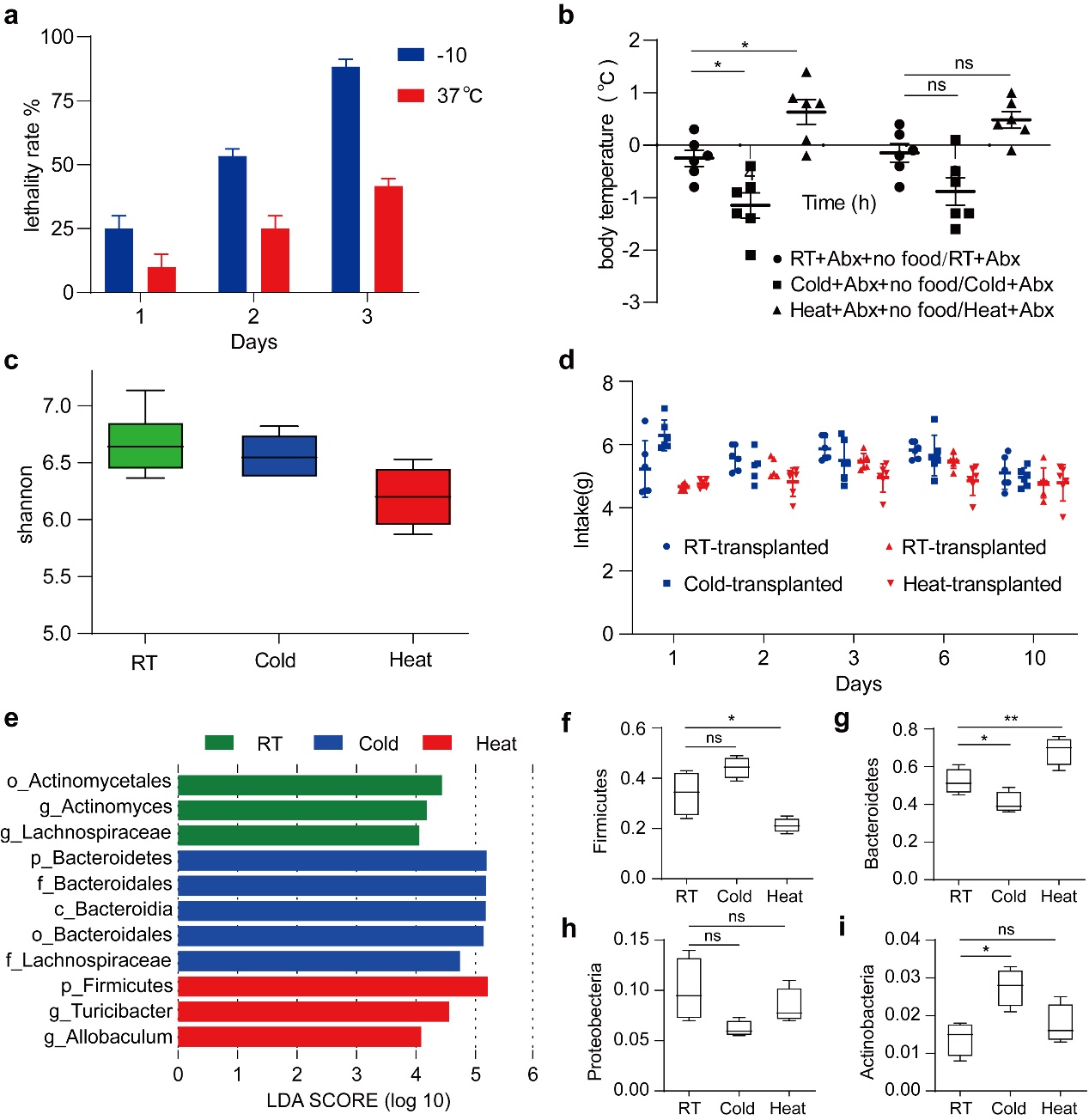


**Fig. S2** (**a**) The lethality rate of mice at -10℃ and 37℃. The experiment was repeated three times (n = 10). (**b**) Changes in the rectal temperature of mice treated with Abx and food fasting after 4 and 8 h of RT, Cold and Heat groups compared to Abx alone. n = 6 (**c**) Food intake by mice treated with Abx at RT-, Cold- and Heat-transplanted. n = 6. (**d**) Alpha diversity measured with the Shannon index of gut microbiota in RT, Cold and Heat mice. (**e**) Comparison of relative abundance of intestinal microbiota of up to 60 days RT, Cold and Heat mice in LDA Effect Size (LEfSe) analysis (LDA score > 4.0). (**f**-**i**) Phylum level relative abundance in rectal. n = 4. All values show mean ± SEM. Significance was calculated using non-paired two-tailed Student’s t test. *p ≤ 0.05, **p ≤ 0.01.


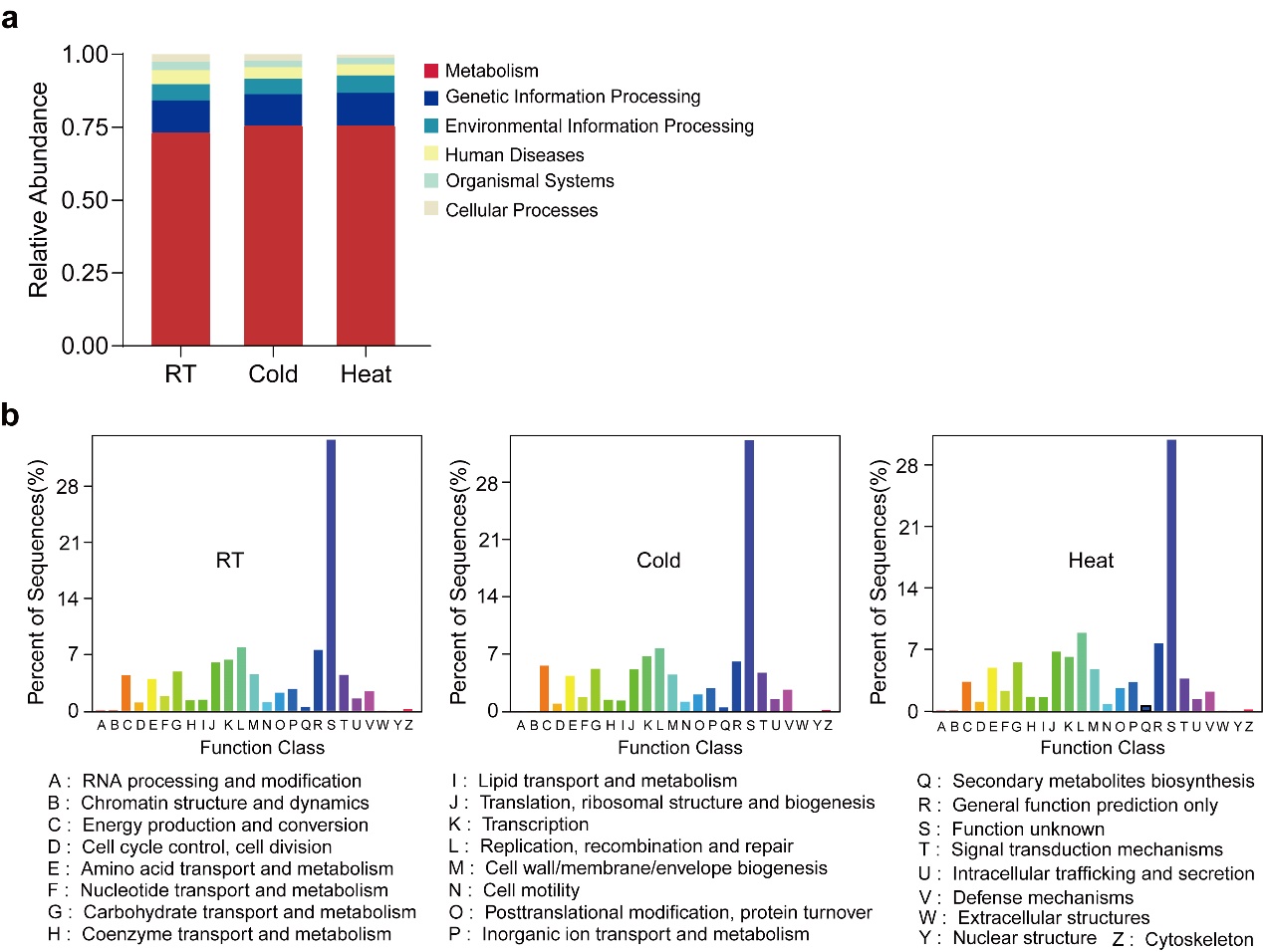


**Fig. S3** (**a**) KEGG pathways at the 1st level based on inferred metagenomes of gut bacteria at RT, Cold and Heat mice. (**b**) The chart of COG Function Classification of gut bacteria in RT, Cold and Heat mice.

Supplementary Table 1

| Gene | Forward primer | Reverse primer |
| --- | --- | --- |
| Slc2a2 | GTCAGCTATTCATCCACATTCAGT | AGCCAAGGTTCCGGTGAT |
| Slc5a1 | CTGGCAGGCCGAAGTATG | TTCCAATGTTACTGGCAAAGAG |
| Sgk1 | GGACTACATTAATGGTGGAGAGC | CTGGCTATTTCAGCTGCGTA |
| Ppara | AGAGCCCCATCTGTCCTCTC | ACTGGTAGTCTGCAAAACCAAA |
| Cidea | TGACATTCATGGGATTGCAGAC | GGCCAGTTGTGATGACTAAGAC |
| Ucp1 | AGGCTTCCAGTACCATTAGGT | CTGAGTGAGGCAAAGCTGATTT |
